# Supplementary material for: ‘But no living man am I’: Bioarchaeological evaluation of the first-known female burial with weapon from the 10th-century-CE Carpathian Basin
Source: PLoS One. 2024 Nov 26;19(11):e0313963. doi: 10.1371/journal.pone.0313963 (PMC11594485; doi:10.1371/journal.pone.0313963)
Supplement: S1 Table — (DOCX) [file pone.0313963.s001.docx]

**S1 Table. Description of joint changes observed on the extant skeletal remains of SH-63.**

| **Bone** | **Anatomical region** | **Joint** | **Description of changes** |
| --- | --- | --- | --- |
| right clavicle | sternal end | sternoclavicular joint | complete remodeling of the surface with marginal osteophytes and osteolytic formations, i.e., macroporosity and pitting with large cavities (ca. 4x5 mm in size with an average depth of 2 mm) present on the superior part of the surface |
| right scapula | glenoid cavity | glenohumeral joint | marginal osteophytes on the inferior, posterior, superior, and anterior margin (a part of the anterior margin and the surface is *post mortem* missing) |
| left scapula | glenoid cavity | glenohumeral joint | marginal osteophytes on the superior and posterior margin (the inferior and a part of the anterior margin is *post mortem* missing) |
|  | acromion | acromioclavicular joint | marginal osteophytes and pitting |
| left humerus | head of humerus | glenohumeral joint | thickening of the anterior part of the margin (the inferior and posterior part of the margin is *post mortem* missing) |
|  | condyle of humerus | elbow joint | small osteophytes (less than 1 mm in size) and pitting on the border between the trochlea and capitulum |
| right ulna | trochlear notch | elbow joint | small marginal osteophytes (less than 1 mm in thickness) on the trochlear notch (both on the sides of the olecranon and coronoid process) |
| left ulna | trochlear notch | elbow joint | small marginal osteophytes (less than 1 mm in thickness) on the trochlear notch on the side of the coronoid process (most of the margin on the side of the olecranon is *post mortem* missing) |
| right radius | head of radius | elbow joint | slight pitting on the articular facet |
| left radius | head of radius | elbow joint | slight pitting on the articular facet |
| left 1st metacarpal | proximal end | carpometacarpal joint of thumb | marginal osteophytes on the sellar surface for the trapezium, especially on the lateral part |
| right coxal bone | acetabulum | hip joint | remodeling of the acetabular rim (supero-posterior part) |
| left coxal bone | acetabulum | hip joint | remodeling of the acetabular rim (supero-posterior part) |
| right femur | head of femur | hip joint | osteophytes on the femoral head around the fovea of ligament of head |
| left proximal foot phalanx (4^th^ or 5^th^) | head of phalanx | proximal interphalangeal joint of the foot | marginal osteophytes on the plantar margin and changes in the normal contour of the joint |
| right proximal foot phalanx (4^th^ or 5^th^) | head of phalanx | proximal interphalangeal joint of the foot | marginal osteophytes on the plantar margin and changes in the normal contour of the joint |
| thoracic and lumbar vertebrae | vertebral body (anular epiphysis) | intervertebral joint | osteophytes on the anterior and/or lateral margin of the endplate (spondylosis deformans) |
| Note: the changes were listed following the criteria described in [89] | | | |
