# Supplementary material for: ‘But no living man am I’: Bioarchaeological evaluation of the first-known female burial with weapon from the 10th-century-CE Carpathian Basin
Source: PLoS One. 2024 Nov 26;19(11):e0313963. doi: 10.1371/journal.pone.0313963 (PMC11594485; doi:10.1371/journal.pone.0313963)
Supplement: S2 Table — (DOCX) [file pone.0313963.s002.docx]

**S2 Table. Description of entheseal changes observed on the extant skeletal remains of SH-63.**

| **Bone** | **Insertion site** | **Muscle/ Ligament** | **Description of changes** | **Score according to [67]** |
| --- | --- | --- | --- | --- |
| right clavicle | rugosity for the deltoid muscle | deltoid muscle | irregularity, slight elevation and enthesophytes | N/a |
|  | impression for costoclavicular ligament | costoclavicular ligament | irregularity, elevation of the surface, and slight enthesophytes | N/a |
| left clavicle | rugosity for the deltoid muscle | deltoid muscle | irregularity, slight elevation of the surface, and enthesophytes | N/a |
| right scapula | infraglenoid tubercle | triceps brachii muscle | enthesophytes and microporosity on the surface | N/a |
| right humerus | crest of the greater tubercle | pectoralis major muscle | irregularity and slight new bone formations | B |
|  | crest of lesser tubercle | latissimus dorsi/ teres major muscle | cortical defect | B |
|  | deltoid tuberosity | deltoid muscle | elevation of the tuberosity, slight erosion and irregularity of the surface | B |
| left humerus | lesser tubercle (distal and lateral part) | subscapular muscle | surface enthesophytes and pitting | B |
|  | crest of greater tubercle | pectoralis major muscle | slight irregularity with a sharp ridge | B |
|  | deltoid tuberosity | deltoid muscle | elevation of the tuberosity, slight erosion and irregularity of the surface | B |
|  | lateral supraepicondylar ridge | brachioradialis and extensor carpi radialis longus muscles | elevation of the ridge and sharp margin | N/a |
|  | lateral epicondyle | common insertion of extensor muscles | protruding and irregular margin and irregular surface | B |
|  | medial epicondyle | common insertion of flexor muscles | protruding and irregular margin and irregular surface | B |
| right ulna | olecranon | triceps brachii muscle | well-developed enthesophytes (≥2 mm in length) with slight surface erosion | C |
|  | tuberosity of ulna | brachialis muscle | irregular surface with erosions | N/a |
|  | supinator crest | supinator muscle | elevation of the crest, erosion and irregularity of the surface, sharp bone ridges inferior to the crest | N/a |
|  | interosseus border | insertion of interosseous membrane and secondary insertion site for the felxor digitorum profundus muscle | thickened and irregular margin | N/a |
| left ulna | olecranon | triceps brachii muscle | well-developed enthesophytes (≤2 mm in length) with slight surface irregularity | B |
|  | tuberosity of ulna | brachialis muscle | irregular surface with erosions | N/a |
|  | supinator crest | supinator muscle | elevation of the crest, erosion and irregularity of the surface, sharp bone ridges inferior to the crest | N/a |
|  | interosseus border | insertion of interosseous membrane and secondary insertion site for the felxor digitorum profundus muscle | thickened and irregular margin | N/a |
| right radius | radial tuberosity | biceps brachii muscle | elevation of the tuberosity, erosion on the majority of the surface, irregular and protruding contour | C |
|  | surface superior and lateral from the anterior oblique line | supinator muscle | elevated and irregular surface | N/a |
| left radius | radial tuberosity | biceps brachii muscle | elevation of the tuberosity, slight erosion on less than half of the surface, slightly irregular and protruding contour | B |
|  | surface superior and lateral from the anterior oblique line | supinator muscle | elevated and irregular surface | N/a |
|  | pronator tuberosity | pronator teres muscle | irregular surface | B |
|  | interosseus border | insertion of interosseous membrane and secondary insertion site for the flexor pollicis longus muscle | slightly elevated and irregular margin | N/a |
| right coxal bone | supra-acetabular groove | rectus femoris muscle (reflected head) | irregular surface with traces of erosion (*post mortem* damaged) | N/a |
|  | ischial tuberosity | semimembranosus, semitendinosus, biceps femoris (long head) muscles | irregular margin and traces of erosion on the surface (*post mortem* damaged) | B |
| right femur | trochanteric fossa | obturator externus, obturator internus muscles | well-developed enthesophytes (≤2 mm in size) | N/a |
|  | gluteal tuberosity | gluteus maximus | irregular surface with erosions | B |
|  | linea aspera (middle medial and lateral lips) | medial lip: adductor longus, adductor magnus, adductor brevis, vastus medialis muscles; lateral lip: vastus lateralis, biceps femoris muscles | irregular surface | B |
| left femur | trochanteric fossa | obturator externus, obturator internus muscles | well-developed enthesophytes (≥2 mm in size) | N/a |
|  | linea aspera (middle medial and lateral lips) | medial lip: adductor longus, adductor magnus, adductor brevis, vastus medialis muscles; lateral lip: vastus lateralis, biceps femoris muscles | traces of irregularity on the surface (*post mortem* damaged) | B |
| Note: N/a – enthesis was not included in [67]. | | | | |
