# Supplementary material for: ‘But no living man am I’: Bioarchaeological evaluation of the first-known female burial with weapon from the 10th-century-CE Carpathian Basin
Source: PLoS One. 2024 Nov 26;19(11):e0313963. doi: 10.1371/journal.pone.0313963 (PMC11594485; doi:10.1371/journal.pone.0313963)
Supplement: S1 Text — (DOCX) [file pone.0313963.s003.docx]

## **Archaeogenetic examination of the skeletal remains**

### **Ancient DNA laboratory work – sample preparation and DNA extraction**

The pre-PCR procedures were conducted in specialized ancient DNA facilities of the Department of Genetics, University of Szeged and the Department of Archaeogenetics, Institute of Hungarian Research, Hungary. Mitogenome data of the sample utilized in this study had already been published ([1]) and now we performed shallow shotgun sequencing. Sample collection was performed with the use of gloves and facemasks to reduce potential contamination with contemporary human DNA. Multiroot tooth from the mandible (shortened as tooth sample), sample from the petrous part of the right temporal bone (shortened as petrosa sample), and a piece from the left humerus (shortened as humerus sample) were obtained from SH-63 for molecular analyses. The selected tooth and bone parts underwent decontamination with bleach, hydrogen-peroxide, and UV treatment, and DNA extraction from the tooth was carried out using the soaking method, a procedure designed to maintain the morphological integrity of the teeth [2]. DNA extraction was carried out from bone powder obtained from the petrosa and humerus samples. 200 mg bone powder aliquot was pre-digested in 1.5 ml of 0.5 M EDTA containing 100 μg/ml Proteinase K for 30 minutes at 48°C to enhance the proportion of endogenous DNA. After centrifugation, the powder was dissolved for 72 hours at 48°C in an extraction buffer composed of 0.45 M EDTA, 250 μg/ml Proteinase K, and 1% Triton X-100. Subsequently, 7.5 ml of binding buffer, consisting of 5 M GuHCl, 90 mM NaOAc, 40% isopropanol, and 0.05% Tween-20, was added to the extract, and DNA purification was conducted using Qiagen MinElute columns. Minimal destructive protocol was carried out on the tooth sample [2]. A pre-digestion step was performed in 3 ml extraction buffer containing 0.5 M EDTA and 100 µg/ml Proteinase K. Samples were incubated with the pre-digestion buffer for 30 min at 48 °C, followed by a 72-h digestion in extraction buffer containing 0.45 EDTA, 250 µg/ml Proteinase K, and 1% Triton X-100 at 48 °C. Then 12 ml of binding buffer containing 5 M GuHCl, 90 mM NaOAc, 40% isopropanol, and 0.05% Tween-20 was added to the extract and DNA was purified on Qiagen MinElute columns. Concentration of DNA extracts was measured with Qubit 3.0 Fluorometer (Invitrogen) using the dsDNA High Sensitivity Assay kit.

### **NGS library construction**

Libraries subjected to partial uracil-DNA-glycosylase (UDG) treatment were prepared following the methodology outlined by Neparáczki and colleagues [3]. In brief, the double-stranded library protocol of Meyer and Kircher [4] with double indexing [5] was used, with the exception that all purifications were done with MinElute columns. Additionally, partial UDG treatment, as described by Rohland and colleagues [6], was applied, but with a modification of reducing the recommended USER and UGI concentrations to half (0.03 U/μl) while extending the incubation time from 30 to 40 minutes. The reaction occurred at 37°C for 40 minutes in a PCR machine with a lid temperature of 40°C.

Subsequently, 1.8 μl of Uracil Glycosylase Inhibitor (UGI, 2U/μl NEB) was added to the reaction, followed by further incubation at 37°C for 40 minutes. Blunt-end repair was conducted by adding 3 μl T4 polynucleotide kinase (10 U/μl) and 1.2 μl T4 DNA polymerase (5 U/μl) to each reaction, with incubation in a PCR machine at 25°C for 15 minutes, followed by another incubation at 12°C for 5 minutes, and cooling to 4°C. After adding 350 μl MinElute PB buffer (QIAGEN) to the reaction, purification was carried out on MinElute columns, and the DNA was eluted in 20 μl EB prewarmed to 55°C. Adapter ligation and adapter fill-in were performed as outlined by Meyer and Kircher [4].

The library preamplification step was excluded, and the libraries were directly double-indexed in a single PCR step after the adapter fill using 19,5 ul Accuprime Pfx Supermix, 1 µl of 10 mg/ml BSA, 1,5 µl of 10 µM indexing P5 and P7 primers and 10,5 µl of DNA template. The PCR cycles were as follows: 95°C for 5 minutes, 12 cycles of 95°C for 15 sec, 60°C for 30 sec, and 68°C for 3 sec, followed by a 5-minute extension at 68°C. The indexed libraries were purified using MinElute columns and eluted in 20 μl EB buffer (Qiagen).

### **DNA sequencing**

DNA extract and library quantities were assessed using the Qubit fluorometric quantification system. The distribution of library fragments was examined using the TapeStation 2200 (Agilent). The endogenous human DNA content of each library was estimated through low-coverage shotgun sequencing, generated on the iSeq 100 platform (Illumina).

### **Bioinformatical processing**

Paired end raw FASTQ sequences were adapter trimmed with cutadapt (version 4.6) using the Illumina universal adapter sequences and default options [7]. Adapter cleaned PE sequences were aligned to the GRCh37 (hs37d5) reference genome by the BWA software (version 0.7.17-r1198) with the MEM algorithm [8]. Aligned reads were quality filtered by the 90% identity to the reference as described in Maróti et al [9]. Duplicate reads were removed by the PICARD tools MarkDuplicate algorithm (version 2.21.3) with the ‘REMOVE_DUPLICATES=true’ option (<http://broadinstitute.github.io/picard>). *Post mortem* damage in the first 5 bases of DNA fragments were assessed by the ATLAS software suite (version 0.9) using the PMD task [10].

### **Genetic sex determination**

Biological sex was first assessed with the method described by Skoglund and colleagues [11]. In addition, we used the Rx method first published by Mittnik and colleagues and modified later by de Flamingh and colleagues, which can give accurate results from several thousands of reads mapping to the human genome [12,13].

References

1. Maár K, Varga GIB, Kovács B, Schütz O, Maróti Z, Kalmár T, et al. Maternal Lineages from 10–11th Century Commoner Cemeteries of the Carpathian Basin. Genes (Basel). 2021;12: 460. doi:https://doi.org/10.3390/ genes12030460

2. Harney É, Cheronet O, Fernandes DM, Sirak K, Mah M, Bernardos R, et al. A minimally destructive protocol for DNA extraction from ancient teeth. Genome Res. 2021;31: 472–483. doi:10.1101/GR.267534.120

3. Neparáczki E, Maróti Z, Kalmár T, Kocsy K, Maár K, Bihari P, et al. Mitogenomic data indicate admixture components of Central-Inner Asian and Srubnaya origin in the conquering Hungarians. PLoS One. 2018;13: 1–24. doi:10.1371/journal.pone.0205920

4. Meyer M, Kircher M. Illumina Sequencing Library Preparation for Highly Multiplexed Target Capture and Sequencing. Cold Spring Harb Protoc. 2010; pdb.prot5448. doi:10.1101/pdb.prot5448

5. Kircher M, Sawyer S, Meyer M. Double indexing overcomes inaccuracies in multiplex sequencing on the Illumina platform. Nucleic Acids Res. 2012;40: 1–8. doi:10.1093/nar/gkr771

6. Rohland N, Harney E, Mallick S, Nordenfelt S, Reich D. Partial uracil – DNA – glycosylase treatment for screening of ancient DNA. Philos Trans R Soc B Biol Sci. 2015;370. doi:10.1098/rstb.2013.0624

7. Martin M. Cutadapt removes adapter sequences from high-throughput sequencing reads. EMBnet.journal. 2011;17: 10–12. doi:10.14806/ej.17.1.200

8. Li H. Aligning sequence reads, clone sequences and assembly contigs with BWA-MEM. Prepr hosted arXiv.org. 2013.

9. Maróti Z, Neparáczki E, Schütz O, Maár K, Varga GIB, Kovács B, et al. The genetic origin of Huns, Avars, and conquering Hungarians. Curr Biol. 2022;32: 2858-2870.e7. doi:10.1016/j.cub.2022.04.093

10. Link V, Kousathanas A, Veeramah K, Sell C, Scheu A, Wegmann D. ATLAS: Analysis Tools for Low-depth and Ancient Samples. Prepr hosted bioRxiv. 2017. doi:10.1101/105346

11. Skoglund P, Storå J, Götherström A, Jakobsson M. Accurate sex identification of ancient human remains using DNA shotgun sequencing. J Archaeol Sci. 2013;40: 4477–4482. doi:10.1016/j.jas.2013.07.004

12. Mittnik A, Wang C-C, Svoboda J, Krause J. A Molecular Approach to the Sexing of the Triple Burial at the Upper Paleolithic Site of Dolní Věstonice. PLoS One. 2016;11: e0163019. doi:10.1371/journal.pone.0163019

13. de Flamingh A, Coutu A, Roca AL, Malhi RS. Accurate sex identification of ancient elephant and other animal remains using low-coverage DNA shotgun sequencing data. G3 Genes, Genomes, Genet. 2020;10: 1427–1432. doi:10.1534/g3.119.400833
