# Supplementary material for: ‘But no living man am I’: Bioarchaeological evaluation of the first-known female burial with weapon from the 10th-century-CE Carpathian Basin
Source: PLoS One. 2024 Nov 26;19(11):e0313963. doi: 10.1371/journal.pone.0313963 (PMC11594485; doi:10.1371/journal.pone.0313963)
Supplement: Attachment — Submitted filename: Response to Reviewers.docx [file pone.0313963.s005.docx]

Dr. Luca Bondioli, PhD

Academic Editor

PLOS ONE

Dear Dr. Bondioli,

We are very thankful for your and the reviewers’ insightful and constructive comments regarding our manuscript entitled “*‘But no living man am I’: Bioarchaeological evaluation of the first-known female burial with weapons from the 10th-century-CE Carpathian Basin*” that was submitted to PLOS ONE (manuscript ID: PONE-D-24-19948). The main text has been modified following the suggestions, and an additional figure (Figure 12) has been created and added. Additionally, new references have been included, and the numbering has been adjusted accordingly. Please note that since these new references were created using Mendeley Desktop, Microsoft Word did not systematically track the updates in the Reference section or their corresponding citations in the main text. Therefore, we have highlighted these new additions with a yellow background color in the tracked version of the manuscript. The revised files have been uploaded to the submission site of the journal. We are sure that you and the reviewers helped us to improve the quality of our manuscript.

Responses to the comments and questions of Reviewer #1

**Reviewer: The research paper, entitled "But no living man am I": Bioarchaeological evaluation of the first-known female burial with weapons from the 10th-century CE Carpathian Basin" by Balázs Tihanyi and co-authors presents the bioarchaeological study of a female burial (No 63), discovered within the 10th century CE century CE cemetery of Sárrétudvari-Hízóföld (eastern Hungary). The tomb yielded a grave goods set that is related to both the female sphere (including ornaments that recurred in other female and subadult tombs at the site) and the male sphere (including weapons and an archery set, which were typical of the Sárrétudvari-Hízóföld male tombs). The study of this funerary context addresses a complex and contentious topic: the identification of female warrior status in past human societies through the examination of funerary evidence. Furthermore, it considers the question of determining an individual's sex and gender based on biological parameters and material culture. The authors are fully aware of the critical nature of the topic and I was pleased to see a lengthy paragraph in the introduction to the paper outlining the state of the art of approaches to the study of gender identity and sex determination from an archaeological and bioarchaeological perspective. In general, this article is well written, the English used is fluid and the approach to the scientific data is critical. In the presentation of the 'palaeopathological' data and subsequent discussions, the authors adopt a nuanced approach, acknowledging the limitations of the sample and the multi-etiology of the lesions under consideration.**

Response: We are grateful for the Reviewer’s insightful comments and suggestions, which have provided invaluable guidance in improving our manuscript. We particularly appreciate your recognition of our approach to the critical topic of sex and gender determination in archaeological contexts, as well as our careful consideration of the limitations inherent in paleopathological analysis. Below, we address each of the Reviewer’s notes in detail.

**Reviewer: However, I would recommend that the authors consider more explicitly that, with the exception of the fractures at the humeroscapular joint, the observed lesions may be more likely to be related to the mature age of the individual and not to a specific activity associated with.**

Response: We agree with the Reviewer that the observed changes are not specific to a particular activity, and their interpretation is indeed limited by the multifactorial etiology, poor bone preservation, and lack of comparative data on other females, with or without weapon in their grave. These limitations are highlighted in the Discussion and conclusions section, in lines 602–621 and 633–650 of the original text. That being said, physical stress remains considered one of the main factors influencing the development of entheseal changes (e.g., Karakostis et al. 2019).

In the case of SH-63, non-mechanical factors such as the advanced age of the individual alone cannot fully explain the development of these changes, particularly given the documented asymmetry in some of the extant entheseal sites and the presence of various changes (as illustrated in Figure 10 of the original text). For instance, the groove at the enthesis of the latissimus dorsi/teres major muscles observed only on the right humerus of SH-63 (illustrated in Figure 10 C of the original text) is characteristic formation more commonly seen in sub-adult individuals, though it also appears in adults. In recent years, a research group led by Olivier Dutour and Hélène Coqueugniot has begun analyzing groove-like formations of the latissimus dorsi/teres major muscle enthesis in sub-adults and young adults using microCT. Their results on the microstructure of these entheses (e.g., Navarre et al. 2023; Navarre et al. 2024), ruled out developmental causes and proposed two hypotheses related to physical activities: a) variations in the adaptive response of the bone in connection with the growth stage, and b) variations in the nature and intensity of biomechanical constraints in connection with age. These findings are promising and offer valuable insights for evaluating entheseal changes.

However, a cautious interpretation remains necessary in the case of SH–63. This is why we used phrases like ‘could have’ or ‘may have had’ in the Discussion and conclusions section when referring the possible lifestyle of SH-63. Despite the recent findings on entheseal changes and existing similarities with the pattern observed in other individuals with weapon equipment, we emphasize that further research is required to fully understand these phenomena and confirm any lifestyle-related implications.

References:

Karakostis, F. A., Jeffery, N., & Harvati, K. (2019). Experimental proof that multivariate patterns among muscle attachments (entheses) can reflect repetitive muscle use. *Scientific Reports*, *9*, 16577. https://doi.org/10. 1038/s41598-019-53021-8

Navarre, G., Dutailly, B., Vanderesse, N., Dutour, O., & Coqueugniot, H. (2024). Effet de l’âge sur l’organisation de l’os trabéculaire en cas d’ostéolyse corticale d’insertion (OCI) Analyse 3D-μCT des changements enthéséaux sur un échantillon ostéoarchéologique juvénile. *Groupe Des Paleopathologistes de Langue Française. Colloque 2024*.

Navarre, G., Dutour, O., & Coqueugniot, H. (2023). Les enthésopathies en creux: développement ou surcharge mécanique? *Groupe Des Paleopathologistes de Langue Française. Colloque 2023*, 20.

**Reviewer: Moreover, the presence of the archer's set in the tomb (an element that, in a post-processualist perspective, could also be indicative of a 'gift' or the exaltation of a status and not of the deposition of the individual personal objects) is worthy of further consideration.**

Response: We agree with the Reviewer concerning the complex symbolism of artifacts in burials. We also acknowledge and align with the methodological considerations of scholars such as Heiko Steuer, Sebastian Brather, and Heinrich Härke, who argue that the evaluation of artifacts does not necessarily reflect the status and lifestyle of the deceased directly. This perspective is highlighted in the Introduction (lines 106–116) and the Discussion and conclusions (lines 742–758) sections of the original text.

In Hungarian archaeology, the relationship between the deceased, the burial customs applied, and the grave goods provided indeed requires further research. However, the most recent overview of the 10^th^-century-CE burials in the Carpathian Basin (referred to as Conquest Period burials) pointed out that artifacts – including clothing elements, jewelry, weapons, and prestigious items – that were specifically crafted and provided for burial and funeral purposes are exceptionally rare in Conquest Period burials (Révész 2020). Furthermore, the investigation of Conquest Period burials primarily focuses on the composition and characteristics of the grave goods and related burial customs, as burial constructions are generally absent (with the exception of traces of wooden coffins), and grave pits do not exhibit significant variation in their size and forms (Révész 2020).

In our case, even if the provided archery equipment did not belong to the personal possessions of SH-63, it does not alter the core findings of our study – namely, that a weapon was found in a 10th-century-CE female grave from the Carpathian Basin, with burial customs similar to those of its male counterparts. This is a significant factor when evaluating the possible social/military status or ‘occupation’ of the individual. However, as stated in the Discussion and conclusions section (lines 742–758 of the original text), this remains an open debate due to methodological problems and the lack of comparative data.

To incorporate your valuable feedback, we have revised and nuanced the Discussion and conclusions sections and we have highlighted the methodological challenges concerning the symbolic nature of grave goods.

Original text (lines 717–720 of the original text):

*“Additionally, the location of the bow remain in the grave suggests a connection with the deceased, as the plate was in a rather functional position, as if ‘she was holding it in her left hand’, such as described by the archaeologist in charge of the excavation of the cemetery [148].”*

Revised text (lines 705–709):

“*While the origin and symbolic significance of these artifacts (e.g., personal items or gifts) remain unclear, the location of the bow remain in the grave suggests a connection with the deceased, as the plate was in a rather functional position, as if ‘she was holding it in her left hand’, such as described by the archaeologist in charge of the excavation of the cemetery [149].*”

Original text (lines 750–758 of the original text):

*“Certainly, one of the most intriguing questions is whether the case can be considered a warrior burial. Unfortunately, at the current research level, this must remain an open debate. First, the term “warrior” involves specific aspects on social and legal levels for which sources other than archaeological and anthropological are also required for identification, but no written data are available concerning warrior women among the Magyars in the 10th century CE. Furthermore, in nomadic tribes of the eastern steppes, similar to the early Magyars, it was common for females to learn how to defend themselves and the livestock to survive [141,142]. While this probably resulted in practicing similar daily activities with males, they were not necessarily considered as dedicated warriors.”*

Revised text (lines 739–749):

*“Certainly, one of the most intriguing questions is whether the case can be considered a warrior burial. Unfortunately, at the current research level, this must remain an open debate. First, the term “warrior” involves specific aspects on social and legal levels for which sources other than archaeological and anthropological are also required for identification, but no written data are available concerning warrior women among the Magyars in the 10th century CE.* *Additionally, the origin and symbolic significance of the artifacts — whether they were the personal items of the deceased or gifts from the community — remain unclear. Furthermore, in nomadic tribes of the eastern steppes, similar to the early Magyars, it was common for females to learn how to defend themselves and the livestock to survive [142,143]. While this probably resulted in practicing similar daily activities with males, they were not necessarily considered as dedicated warriors”*

Reference:

Révész, L. (2020). *A 10–11. századi temetők regionális jellemzői a Keleti-Kárpátoktól a Dunáig*. Szegedi Tudományegyetem Régészeti Tanszéke–Magyar Tudományos Akadémia Régészeti Intézete–Magyar Nemzeti Múzeum–Martin Opitz Kiadó.

**Reviewer: Additionally, as proposed below, I would like the authors to extend the comparison between the bioarchaeological evidence gathered in this study on individual SH 63 and that available not only on the other individuals with weapons in the tombs, but also on the other female individuals and, in general, for the necropolis as a whole. This would help to avoid forcing a connection (tombs with weapons) that could produce flawed observations.**

Response: We appreciate the Reviewer's suggestion to extend the comparison. We agree that avoiding a forced connection between tombs with weapons is essential to prevent potentially flawed conclusions.

The primary limitation of our interpretation lies in the uniqueness of SH-63 and the current lack of comparative data, as highlighted in lines 759–760 of the original text. Although data on pathological and supposed activity-related changes in males with and without weapons from the Sárrétudvari–Hízóföld cemetery has increased over the last decade (e.g., Berthon et al. 2015; Tihanyi et al. 2015; Berthon 2019; Berthon et al. 2019; Tihanyi et al. 2020; Tihanyi 2020), similar data on female individuals remains highly limited. The absence of data on females highlights the need for extensive future research to build a comprehensive database, including data on female individuals from various 10th-century-CE series of the Carpathian Basin. At present, comparison is only possible with data published in the 1990s (e.g., Pálfi 1992; 1993; Pálfi & Dutour 1996; Pálfi 1997), and we must acknowledge that methodologies, particularly in the diagnosis of various pathological changes, have evolved over the intervening decades.

In the Sárrétudvari–Hízóföld series, six cases with possible traces of osteoporosis were described, five of which were identified as adult females, while one individual was classified as indeterminate adult (Pálfi 1993; 1997). In contrast, no osteoporosis-related changes were documented in adult males. In addition, traumatic lesions, which were frequently observed in the series, were typically associated with males, with only a few cases registered in females. This suggests differences in lifestyle between the sexes (e.g., Pálfi 1992; 1993; Pálfi & Dutour 1996). Similarly, entheseal and joint changes were predominantly found in males (e.g., Pálfi 1993; Pálfi & Dutour 1996; Pálfi 1997), with entheseal changes of the upper limb bones reported in 21 males but only in one female (Pálfi 1997).

Following the Reviewer’s suggestion, we have extended the relevant parts of the Discussion and conclusions section. However, we have done so cautiously, addressing the above-described methodological issues and focusing on the trends observed by earlier scholars in the Sárrétudvari–Hízóföld series.

Changes applied to the Discussion and conclusions section:

1. Osteoporosis (including changes requested by Reviewer #2):

Original text (lines 547–555 of the original text):

"*Several antemortem features are present on the skeletal remains of SH-63. Firstly, we observed that the bones are much lighter than in the case of other skeletons from the Sárrétudvari–Hízóföld series, possibly due to osteopenia, a loss of bone mass. Particularly, bone fragility, traces of a reduced trabecular system in the vertebrae, an increased diameter of the medullary cavity in the long bones, thinning of cortical bone in both the skull and postcranial elements, and antemortem bone fractures suggest the presence of osteoporosis in the skeleton (e.g., [165,179–182]). The presence of osteoporosis, described as a disease that typically affects older women [165,180], indirectly supports the results concerning the sex determination of SH-63.*”

Revised text (lines 500–521):

*“Several antemortem features are present on the skeletal remains of SH-63.* *Firstly, we observed the lightweight nature of the bones, which could indicate osteopenia, a loss of bone mass. Specifically, bone fragility, traces of a reduced trabecular system in the vertebrae, an increased diameter of the medullary cavity in the long bones, thinning of cortical bone in both the skull and postcranial elements, and antemortem bone fractures may be associated with the presence of osteoporosis in the skeleton (e.g., [166,174–177]). The presence of osteoporosis, described as a disease that typically affects older women [166,175],* *could indirectly support the results concerning the sex determination of SH-63. However, the assessment of the registered features is limited for two main reasons. Firstly, diagnosing osteoporosis is complex and requires consideration of several methodological factors, including the use of quantitative methods (e.g., radiometry or histomorphometry) [178,179]. As no radiological or histological investigations could be conducted during the analysis of SH-63, there is no such evidence for a definitive diagnosis. Secondly, poor bone preservation complicates the detailed discussion of diagenetic changes in relation to bone loss. Nevertheless, it is unlikely that diagenetic changes alone account for the observed changes, and the presence of pathological conditions, including osteoporosis, should be considered, especially given the antemortem bone fractures found in the skeleton of SH-63. In earlier studies focusing on the paleopathological investigation of the Sárrétudvari–Hízóföld series, six cases with possible traces of osteoporosis were registered, including five adult females and one adult of indeterminate sex. In contrast, no osteoporosis-related changes were observed in adult males (e.g., [151,180]). However, comparative analysis with currently available data at the populational level is constrained by methodological changes in paleopathological diagnostics over the subsequent decades.”*

2. Traumatic lesions:

Original text (lines 608–613 of the original text):

*“Traumas and bone fractures are also known in other skeletons from the Sárrétudvari–Hízóföld series (e.g., [143,144,154,157]). Particularly, polytraumatism and fractures of the upper limb occurred in the highest number in the group of individuals buried with weapons and/or horse riding equipment, suggesting that lifestyle –including horse riding– is responsible for the high number of traumas in the Sárrétudvari–Hízóföld series (e.g., [143,144]).”*

Revised text (lines 561–569):

*“Traumas and bone fractures are also known in other skeletons from the Sárrétudvari–Hízóföld series (e.g., [144,145,155,158]).* *Previous paleopathological studies of the series have highlighted that while fractures and other traumas are common among males, such changes are exceptionally rare in females, suggesting differences in lifestyle between the sexes (e.g., [78,151,186]). Particularly, polytraumatism and fractures of the upper limb occurred in the highest number in the group of individuals buried with weapons and/or horse riding equipment, suggesting that lifestyle –including horse riding– is responsible for the high number of traumas in the Sárrétudvari–Hízóföld series (e.g., [144,145]).”*

3. Entheseal and joint changes:

Original text (lines 625–629 of the original text):

*“Comparative analysis is also limited, as this is the first known female burial with weapon equipment from the 10^th^-century-CE Carpathian Basin. Nevertheless, we found asymmetry in the occurrence and characteristics of entheseal changes at the insertion sites of the main muscles of the upper limb bones, suggesting the influence of physical stress on their development.”*

Revised text (579–590):

*“Comparative analysis is also limited, as this is the first known female burial with weapon equipment from the 10^th^-century-CE Carpathian Basin.* *Previous studies on paleopathological and supposed activity-related skeletal changes in the series have revealed a disparity between the sexes in the distribution of these alterations, with entheseal and joint changes predominantly found in males, indicating differences in lifestyle [78,151,180]. For instance, entheseal changes in the upper limb bones were reported in 21 males but only in one female (e.g., [180]). However, comparative analysis with earlier findings is limited due to methodological changes in the registration and evaluation of activity-related changes over the subsequent decades.*

*Nevertheless, we found asymmetry in the occurrence and characteristics of entheseal changes at the insertion sites of the main muscles of the upper limb bones, suggesting the influence of physical stress on their development.”*

References:

Berthon W, Rittemard C, Tihanyi B, Pálfi G, Coqueugniot H, Dutour O. Three-dimensional microarchitecture of entheseal changes: Preliminary study of human radial tuberosity. *Acta Biol Szeged*. 2015;59: 79–90.

Berthon W. *Bioarchaeological analysis of the mounted archers from the Hungarian Conquest period (10th century): Horse riding and activity-related skeletal changes*. École Pratique des Hautes Études, PSL University–University of Szeged. 2019.

Berthon W, Tihanyi B, Kis L, Révész L, Coqueugniot H, Dutour O, et al. Horse riding and the shape of the acetabulum: Insights from the bioarchaeological analysis of early Hungarian mounted archers (10th century). *Int J Osteoarchaeol*. 2019;29: 117–126. doi:10.1002/oa.2723

Pálfi G. Traces des Activités sur les Squelettes des Anciens Hongrois. *Bull Mem Soc Anthropol Paris*. 1992;3–4: 209–231.

Pálfi G. *Maladies, activites et environnements de populations anciennes en Europe Centrale et Occidentale: Approche de paleopathologie comparee*. Universite de Provence–József Attila Tudományegyetem. 1993.

Pálfi G, Dutour O. Activity-induced skeletal markers in historical anthropological material. *Int J Anthropol*. 1996;11: 41–55. doi:10.1007/BF02442202

Pálfi G. Maladies dans l’Antiquité et au Moyen-Âge. Paléopathologie comparée des anciens Gallo-Romains et Hongrois. *Bull Mem Soc Anthropol Paris*. 1997;9: 1–206.

Tihanyi B, Bereczki Z, Molnár E, Berthon W, Révész L, Dutour O, et al. Investigation of Hungarian Conquest Period (10th c. AD) archery on the basis of activity-induced stress markers on the skeleton – preliminary results. *Acta Biol Szeged*. 2015;59: 65–77. Available: http://www2.sci.u-szeged.hu/ABS/2015/Acta HP/5965.pdf

Tihanyi B, Berthon W, Kis L, Váradi OA, Dutour O, Révész L, et al. “Brothers in arms”: Activity-related skeletal changes observed on the humerus of individuals buried with and without weapons from the 10th-century CE Carpathian Basin. *Int J Osteoarchaeol*. 2020;30: 798–810. doi:10.1002/oa.2910

Tihanyi B. *10. századi fegyveresek aktivitás okozta szkeletális elváltozásainak összehasonlító vizsgálata. A fegyvermelléklet adás szokásának bioarchaeológiai megközelítése*. University of Szeged. 2020.

**Reviewer: From a methodological and analytical perspective, it is my contention that the application of further investigative tools, such as bone histology, would enhance the reliability of the conclusions drawn. This would entail the verification of phenomena such as osteopenia, osteoporosis and bone fragility, and the distinction between these and phenomena of diagenetic bioerosion. Furthermore, radiographic examination of the fracture injuries would enable a more detailed investigation of the state of bone remodelling and healing. It would also be of interest to ascertain whether there are any differences between the sexes and between individuals of the same sex in terms of nutrition (carbon and nitrogen isotopes) and origin (oxygen and strontium isotopes, for example).**

Response: We appreciate the Reviewer's insightful suggestion regarding the potential benefits of applying additional investigative tools. We agree that such analyses could significantly deepen our understanding of SH-63 and the broader Sárrétudvari population. However, due to the limitations of our current study, the skeletal remains of SH-63 were only examined using the methods described, and we were unable to conduct the recommended investigations.

Looking ahead, we hope that this study will pave the way for future research opportunities, enabling us to secure funding to apply these analytic methods to SH-63 and other comparable cases. Nevertheless, it is important to consider the sample requirements and the potential impact of poor bone preservation on the results of these analyses. Additionally, the sampling process may result in the destruction of bone elements needed for further study. These factors will require careful consideration in the planning of any follow-up investigation.

**Reviewer: Finally, I would recommend to provide images of a higher quality, as the low resolution of the figures submitted for review made it challenging to accurately compare the lesions described in the text with their photographic documentation.**

Response: Thank you for bringing this issue to our attention. We apologize for the difficulties caused by the low resolution of the figures during the review process. We encountered the same issue when uploading the material to the submission system, as the images were compressed, resulting in lower quality. However, the images can be viewed in their original resolution by accessing the link provided in the upper-right corner of the related pages. We believe this may be the source of the problem, as the images passed the journal's technical check, and other reviewers did not report similar concerns. We will ensure that all figures are in their full resolution and double-check that the final submission retains the highest possible image quality.

**Reviewer: Below are a few more detailed observations:**

Response: We appreciate the Reviewer's detailed suggestions. The following changes have been made to the original text based on these observations.

**Reviewer: lines 47-49: Although the discussion makes it clear that the hypotheses regarding the presence of the fractures and other bone lesions (the latter being of a multi-etiological nature) may be connected either to the activities carried out in life by the woman (use of the bow, horse-riding) or to other idiosyncratic and/or ontological factors, the abstract places too much emphasis on seeking a connection between the evidence found on the remains of the female with weapons and the other armed males in the necropolis. This connection may lead to a flawed interpretation of the data, which has not been compared with the other unarmed individuals in the necropolis, both male and female. It would be advisable for the authors to consider making this sentence more nuanced.**

Response: We have rephrased the sentence as follows:

Original text (lines 47–49 of the original text):

*“In addition, the pattern of pathological and activity-related changes observed on the bones aligns with features found in the skeletal remains of other individuals buried with weapons, suggesting possible similarities in lifestyle.”*

Revised text (lines 47–49):

*“**In addition, the pattern of pathological and supposed activity-related changes observed on the bones may have resulted from regular physical activity during her lifetime.”*

**Reviewer: lines 61-62: When referring to archaeology, I would always use the term gender and not sex.**

Response: We appreciate the Reviewer's suggestion and agree that "gender" is typically the more appropriate term in archaeological contexts. However, in this instance, we deliberately used the term "sex" to highlight a specific methodological issue, where the distinction between biological sex and gender identity is often blurred, particularly in publications where sex is inferred from grave goods. The sentence in question draws attention to this issue, which we further elaborate on in the subsequent sections of the introduction.

**Reviewer: Additionally, the expression 'classic archaeology' can be confused with 'classical', whereas the authors here refer to 'traditional archaeology'.**

Response: In light of Reviewer's comment, we have replaced the term "classic archaeology" with "traditional archaeology" to avoid any potential confusion.

**Reviewer: lines 66-67: The term 'classical anthropology' should be changed to 'traditional anthropology'. It would be advisable to revise the sentence, as the methods of traditional anthropology, based on morphology and morphometrics, although less expensive than DNA techniques, are no simpler.**

Response: In response to the Reviewer's comment, we have replaced the term "classical anthropology" with "traditional anthropology" and removed the word "simplicity" from the sentence.

**Reviewer: line 84: Please, add to the bibliography on the use of amelogenin in archaeological contexts the article by Lugli and colleagues (2019) ' Enamel peptides reveal the sex of the Late Antique 'Lovers of Modena'.**

Response: The suggested reference by Lugli et al. (2019) has been added to the bibliography.

**Reviewer: linee 219-221: Please, provide a more detailed description of the composition of the sample and how the number of males and females has changed since the previous analyses. Out of a total of 162 adults, the previous analyses identified 70 females and 85 males (155 sexually determined and 7 non-determined), while the sex was subsequently confirmed for 52 females and 69 males. Has the number of undetermined people therefore increased?**

Response: Thank you for your observation. We acknowledge the need to clarify the changes in the composition of the sample and the methods used. Decades after the initial biological sex determination using the method of Éry and colleagues (Éry et al. 1963), an independent investigation was conducted by our colleague, Dr. William Berthon (Berthon 2019), using Brůžek’s morphological method (Brůžek 2002) and DSP2 (Brůžek et al. 2017).

In the majority of unconfirmed cases, the remaining original males and females were deemed of indeterminate sex or non-observable, primarily due to two methodological reasons: a) the results did not meet the required thresholds for a definitive male or female determination; or b) analysis was not feasible due to poor preservation or complete absence of the pelvic bones. The method by Éry and colleagues, which involves the skull, pelvis, and femur, was applicable even when the pelvic bones were unsuitable for analysis.

However, in six cases, opposite results were obtained with one (in four cases; the other method was either non-applicable or yielded an indeterminate result) or with both methods (in two cases) during the reassessment of biological sex. Although we plan to conduct a methodological project to explore the factors behind these discrepancies, this remains a controversial issue for now, as no archeogenetic data is available for these individuals. In addition, these burials contained only grave goods that were either gender-neutral or absent altogether.

Subsequently, archeogenetic investigations were conducted on a representative number of samples from the series, initially focusing on mitogenome data (Maár et al. 2021) and later on whole genome data (Maróti et al. 2022). In the cases included in these projects, no conflict was detected between the anthropological and archeogenetic sex determination data, except for the individual studied in our manuscript.

To enhance clarity, the following revisions have been made in response to the comments from Reviewer #1 and Reviewer #3:

Original text (lines 219–221 of the original text):

*“Among these individuals, 101 were sub-adults and 162 were adults. Besides, 70 females and 85 males were identified applying anthropologic methods [155]. Later, during the reassessment of age at death and sex of the SH individuals, the biological sex of 52 females and 69 males was confirmed using other anthropological and archaeogenetic methods [144].”*

Revised text (lines 218–223):

*“Among these individuals, 101 were sub-adults and 162 were adults**. Initially, 70 females and 85 males were identified using anthropological methods that focused on both the skull and postcranial skeleton [155]. Later, a reassessment of the age at death and sex of the SH individuals confirmed the biological sex of 52 females and 69 males through additional anthropological methods focusing only on the pelvis [144] and archaeogenetic analyses of a representative sample [113,146].”*

References:

Berthon, W. (2019). *Bioarchaeological analysis of the mounted archers from the Hungarian Conquest period (10th century): Horse riding and activity-related skeletal changes*. École Pratique des Hautes Études, PSL University–University of Szeged.

Brůžek, J. (2002). A Method for Visual Determination of Sex, Using the Human Hip Bone. *American Journal of Physical Anthropology*, *117*, 157–168. https://doi.org/10.1002/ajpa.10012

Brůžek, J., Santos, F., Dutailly, B., Murail, P., & Cunha, E. (2017). Validation and reliability of the sex estimation of the human os coxae using freely available DSP2 software for bioarchaeology and forensic anthropology. *American Journal of Physical Anthropology*, *164*, 440–449. https://doi.org/10.1002/ajpa.23282

Éry, K., Kralovánszky, A., & Nemeskéri, J. (1963). Történeti népességek rekonstrukciójának reprezentációja. *Antropológiai Közlemények*, *7*, 41–90.

Maár, K., Varga, G. I. B., Kovács, B., Schütz, O., Maróti, Z., Kalmár, T., Nyerki, E., Nagy, I., Latinovics, D., Tihanyi, B., Marcsik, A., Pálfi, Gy., Bernert, Z., Gallina, Z., Varga, S., Költő, L., Raskó, I., Török, T., & Neparáczki, E. (2021). Maternal Lineages from 10–11th Century Commoner Cemeteries of the Carpathian Basin. *Genes*, *12*, 460. https://doi.org/https://doi.org/10.3390/ genes12030460

Maróti, Z., Neparáczki, E., Schütz, O., Maár, K., Varga, G. I. B., Kovács, B., Kalmár, T., Nyerki, E., Nagy, I., Latinovics, D., Tihanyi, B., Marcsik, A., Pálfi, Gy., Bernert, Z., Gallina, Z., Horváth, C., Varga, S., Költő, L., Raskó, I., … Török, T. (2022). The genetic origin of Huns, Avars, and conquering Hungarians. *Current Biology*, *32*(13), 2858-2870.e7. https://doi.org/10.1016/j.cub.2022.04.093

**Reviewer: line 259: Please, provide a rationale for the authors' determination of the percentage of exhibit preservation as greater than 50 per cent, and include a reference. line 263: As above. Please, add reference.**

Response: We appreciate the Reviewer’s insightful comment, which highlighted a mistake in the data provided related to our determination of preservation. We based our assessment on the categorization system described in Spekker et al. 2024, which is widely utilized by Hungarian anthropologists for analyzing skeletal remains. According to this system, the completeness of the skeleton is ranked as follows: 1) almost complete – more than 80% of the skeleton is extant; 2) relatively complete – 50–80% of the skeleton is extant; and 3) very incomplete – less than 50% of the skeleton is extant. The preservation of the skeleton is ranked as: 1) well-preserved – minimal overall post-mortem damage to the bone surfaces; 2) fairly preserved – moderate overall post-mortem damage to the bone surfaces; and 3) poorly preserved – significant overall post-mortem damage to the bone surfaces.

In light of this comment, we have corrected the relevant section in the Materials and Methods and included the appropriate reference as follows:

Original text (lines 258–265 of the original text):

*“The skeletal remains of the individual (SH-63) recovered from the grave are in a poor condition (Fig 2A). The cranium is relatively complete (more than 50% of the bones are present) and fairly preserved, with moderate damage on the surfaces. However, the facial skeleton is highly incomplete and poorly preserved, with only the mandible, fragments of the two maxillae, and the left zygomatic bone being present. The postcranial skeleton is relatively complete (more than 50% of the bones are present) but poorly preserved, especially the bones of the spine (e.g., vertebral bodies), thoracic wall (e.g., sternum), and pelvis (e.g., sacrum and ilium), mostly consisting of bone fragments from these regions.”*

Revised text (lines 259–266):

*“**The skeletal remains of the individual (SH-63) recovered from the grave are in poor condition (Fig 2A). According to the criteria* *described by Spekker et al. [164], the cranium is relatively complete (50–80% is extant) and fairly preserved, with moderate damage to the surfaces. However, the facial skeleton is highly incomplete and poorly preserved, with only the mandible, fragments of the two maxillae, and the left zygomatic bone present. The postcranial skeleton is relatively complete (50–80% of the bones are extant) but poorly preserved, especially the bones of the spine (e.g., vertebral bodies), thoracic wall (e.g., sternum), and pelvis (e.g., sacrum and ilium**), which are mostly represented by of bone fragments.”*

Reference:

Spekker, O., Attila Kiss, P., Kis, L., Király, K., Varga, S., Marcsik, A., Schütz, O., Török, T., Hunt, D. R., & Tihanyi, B. (2024). White plague among the “forgotten people” from the Barbaricum of the Carpathian Basin – Cases with tuberculosis from the Sarmatian-period (3rd–4th centuries CE) archaeological site of Hódmezővásárhely–Kenyere-ér, Bereczki-tanya (Hungary). *PLoS ONE*, *19*(1 January), 1–29. https://doi.org/10.1371/journal.pone.0294762

**Reviewer: line 269: Please, add reference for thickness of cranial bones.**

Response: The general conditions, including bone thickness, were recorded following the methodologies described in Buikstra and Ubelaker 1994, Ortner 2003, and White et al. 2012. We have added the relevant references to the manuscript accordingly.

References:

Buikstra, J. E., & Ubelaker, D. H. (1994). *Standards for data collection from human skeletal remains*. Arkansas Archaeological Survey.

Ortner, D. J. (2003). *Identification of pathological conditions in human skeletal remains*. Academic Press.

White, T. D., Black, M. T., & Folkens, P. A. (2012). *Human Osteology* (Third edit). Academic Press.

**Reviewer: line 272: Please, add reference for age estimation based on auricular surface or symphysial surface.**

Response: We appreciate the Reviewer’s suggestion regarding the addition of references for age estimation based on the auricular or symphysial surface. However, in the corresponding section, we explain that *post mortem* damage of these areas prevented the use of these methods. Therefore, including references for these techniques may lead to confusion for the readers.

**Reviewer: Furthermore, the authors do not discuss the estimation of age at death based on the wear of the occlusal surfaces of the dental enamel. It is unclear why this parameter was not considered for the individual SH63.**

Response: We acknowledge that age-at-death estimation based on tooth wear was not addressed in our manuscript. As highlighted by several scholars, tooth wear is influenced by a variety of external and internal factors, including diet and tooth morphology (e.g., White et al. 2012). Unfortunately, we lack specific data for SH-63 regarding these factors, which are critical for accurate scoring, as well as detailed comparative data for the Sárrétudvari–Hízóföld population. Therefore, scoring and discussing this marker could introduce methodological biases and potentially obscure rather than clarify our findings on age-at-death estimation.

Reference:

White, T. D., Black, M. T., & Folkens, P. A. (2012). *Human Osteology* (Third edit). Academic Press.

**Reviewer: lines 357-364: I would move the ethical statement to the appropriate space in the submission form.**

Response: We appreciate the Reviewer’s suggestion regarding the placement of the ethical statement. We have formatted the manuscript and related documents, including the ethical statement, in accordance with the requirements outlined in the PLOS ONE author guidelines.

**Reviewer: lines 404-409: Please, add reference for all the changes in bone anatomy described in this paragraph.**

Response: We have added the appropriate references to the paragraph detailing the changes in bone anatomy to support our findings.

**Reviewer: lines 418-422: Please, provide a detailed account of the methodology employed by the authors to distinguish this type of lesion from post-depositional and diagenetic alterations. Additionally, please include a comprehensive bibliography. As previously stated, these types of alterations were deemed worthy of investigation through the analysis of bone cortical histology.**

Response: We appreciate the Reviewer’s request for a detailed account of our methodology for distinguishing between bone lesions and post-depositional or diagenetic alterations. Our investigation involved a macromorphological analysis of the skeletal remains, utilizing standard and widely accepted osteological and paleopathological methods, as outlined in the Material and Methods section. We concur that histological analysis could yield valuable data for evaluating changes in the bones. However, this invasive approach would necessitate the destruction of bone material, which could lead to the loss of other potentially significant information. As indicated in lines 415–422 and illustrated in Figure 5, the changes referenced by the Reviewer form part of a lesion complex that includes exostoses and angulation of the proximal bone end. Based on existing literature on post-depositional and pathological changes, even if some lesions have a *post mortem* origin – which we acknowledge in the Discussion and conclusions (lines 557–558 of the original text) section – these changes must have developed antemortem (e.g., Schultz 2001; 2003; Ortner 2003; Galloway 2014). Thus, while some *post mortem* alterations may limit our observations, they do not compromise the diagnostic conclusions drawn for the healed antemortem trauma.

In light of the Reviewer’s feedback, we have modified the Discussion and conclusions sections for greater nuance, and we have added references related to post-depositional changes as follows:

Original text (lines 557–558 of the original text):

“*Although the post mortem loss of the right humeral head limits the observations, the changes described on the humerus are related to a healed antemortem trauma, particularly a possible two-part neck fracture caused by indirect trauma (e.g., [89,165,166,183–185]).*”

Revised text (lines 523–527):

*“**Although post mortem changes, such as the loss of the right humeral head, limit the observations [4,166,181–183], the changes described on the humerus, including the exostoses and angulation of the proximal bone end, are related to a healed antemortem trauma, particularly a possible two-part neck fracture caused by indirect trauma (e.g., [90,166,168,183–185]).”*

References:

Galloway, A. (2014). The upper extremity. In V. L. Wedel & A. Galloway (Eds.), *Broken Bones. Anthropological Analysis of Blunt Force Trauma. Second Edition* (pp. 195–244). Charles C. Thomas.

Ortner, D. J. (2003). *Identification of pathological conditions in human skeletal remains*. Academic Press.

Schultz, M. (2001). Paleohistopathology of Bone: A New Approach to the Study of Ancient Diseases. *Yearbook of Physical Anthropology*, *44*, 106–147.

Schultz, M. (2003). Light Microscopic Analysis in Skeletal Paleopathology. In *Identification of Pathological Conditions in Human Skeletal Remains* (pp. 73–108). Academic Press.

**Reviewer: line 541: Please, add the reference to ' by Skoglund and colleagues'.**

Response: We have added the appropriate reference to the Rx method and to the work of Skoglund and colleagues in the manuscript.

**Reviewer: lines 633-637: Although the authors acknowledge the multi-etiology of the lesions identified on the remains of SH 63, they do not explicitly address the possibility that these lesions may be caused by physiological and degenerative-ontological processes within the skeleton, rather than being strictly related to the lifestyle of the individual. The authors do not provide a precise age at death for the individual, although they do hypothesise osteopenia and osteoporosis (post-reproductive age?).**

**It would be advisable for the authors to consider the influence of the individual's age at death when expanding this part of the discussion.**

**This factor should also be considered when comparing injury data from other individuals who were armed. Rather than considering similarities in lifestyle, can we posit that they were compatible individuals in terms of age at death?**

Response: We agree with the Reviewer that advanced age and the associated degenerative processes significantly contribute to the development of activity-related changes, including entheseal and joint changes, as highlighted in the Discussion and conclusions section (lines 604–605 of the original text). However, it is important to note that repetitive physical loading remains a central factor in this process. While we acknowledge that the advanced age of SH-63 could contribute to the observed changes, it cannot solely account for them, particularly given the documented asymmetry at certain entheseal sites and the presence of specific alterations. To provide a more nuanced interpretation, we have included the influence of age at death among the factors that limit our interpretation of the activity-related changes observed on the skeleton of SH-63.

Original text (lines 634–637 of the original text):

*“Although the interpretation is highly limited due to the poor state of preservation of the bones and the multifactorial etiology of the registered changes, we can assume that physical stress and the possible lifestyle of the individual were among the main contributors to their development.”*

Revised text (lines 619–625):

“*The interpretation is highly limited due to the poor state of preservation of the bones, the possible advanced age of the individual, and the multifactorial etiology of the registered changes. While advanced age and associated degenerative processes could contribute to the observed changes, it is essential to consider that repetitive physical stress and lifestyle factors also play a significant role. The presence of specific alterations and asymmetry at certain entheseal sites suggests that physical stress related to the individual’s lifestyle remains a key factor in these changes.*”

Regarding the estimation of the individual’s age at death, we did not provide a precise age due to the inherent challenges in osteological research, particularly for SH-63. As noted in the Material and Methods section (lines 270–272 of the original manuscript), *“providing a more precise age-at-death is unachievable due to the severe post mortem damage or complete absence of most bone parts used for age estimation (e.g., auricular surface or symphysial surface).”* Consequently, any further discussion or attempts at precise estimation would be speculative without adequate methodological support.

Extensive investigations have been conducted on traumatic lesions in individuals from the Sárrétudvari–Hízóföld cemetery (e.g., Pálfi 1993; 1997; Berthon et al. 2021). None of these studies identified correlations between specific age categories and the presence of injuries. However, they did suggest differences in lifestyle between the sexes (Pálfi 1993; 1997) and indicated a potential link to specific activities, including horse riding (Berthon et al. 2021). Therefore, while age-related factors are acknowledged, the existing data support the idea that the emphasis remains on similarities in lifestyle when comparing injury across individuals.

References:

Berthon, W., Tihanyi, B., Váradi, O. A., Coqueugniot, H., Dutour, O., & Pálfi, Gy. (2021). Riding for a fall: Bone fractures among mounted archers from the Hungarian Conquest period (10th century CE). *International Journal of Osteoarchaeology*, *31*(5), 926–940. https://doi.org/10.1002/oa.3010

Pálfi, Gy. (1993). *Maladies, activites et environnements de populations anciennes en Europe Centrale et Occidentale: Approche de paleopathologie comparee*. Universite de Provence–József Attila Tudományegyetem.

Pálfi, Gy. (1997). Maladies dans l’Antiquité et au Moyen-Âge. Paléopathologie comparée des anciens Gallo-Romains et Hongrois. *Bulletins et Mémoires de La Société d’Anthropologie de Paris*, *9*(1–2), 1–206.

Responses to the comments and questions of Reviewer #2

We would like to express our sincere gratitude to the Reviewer for their invaluable comments and suggestions, which have significantly improved our manuscript. Below, we address each of the Reviewer's notes in detail.

**Reviewer: As indicated in the article, multiple burials are known from this time period where women were buried accompanied by arrowheads. They have only been interpreted differently by previous scholars (as amulets), due to the lack of additional gear (and likely a different mentality in interpretation in previous times). The presence of the bow plate in combination with the arrowhead and possibly quiver remnants is a good indication that the arrowhead would be a utility item/weapon rather older interpretations for the arrowheads. However, this is the only weapon found in the grave, so it is not plural, and if other graves also contain arrowheads, is it really the first? You could consider rephrasing 'first ...with archery gear'**

Response: We agree with the Reviewer’s reasoning that elements of archery equipment (i.e., bow, quiver, and arrowheads) should be considered as a complete set, and we have adjusted our terminology accordingly. Specifically, we have changed the plural form of “weapon(s)” to the singular form when referring to the inventory of SH-63. This adjustment has been made in the Title (line 5 in the original text), Abstract (lines 43 and 51), Introduction (line 197), and Discussion and conclusions (lines 722, 731, 739, and 761) section.

We also acknowledge the Reviewer’s suggestion to use the phrase ‘first … with archery gear’ as a more neutral term. However, we believe that the phrase ‘first … with weapon’ is still suitable and accurately reflects the significance of this finding for the following reasons:

a) Contextual evidence of the bow and arrow as a primary weapon: Both written sources and archaeological evidence from inside and outside the Carpathian Basin, including not only burials with archery equipment but also supposed “Hungarian-type” arrowheads found in destruction levels of settlements and burials (where they were lodged in vertebrae or other bones) (e.g., Schulze-Dörrlamm 2023), suggest that the bow and arrow were the primary weapons of the Hungarians in the 10^th^ century CE. In addition, Hungarians were trained in horseback archery from childhood. This is also reflected in the archaeological data at the micro regional level as in most cemeteries the number of burials containing archery equipment significantly exceeds those with melee weapons. Furthermore, the distribution of graves with archery equipment is concentrated in certain regions and sites (e.g., Révész 2020), such as the Sárrétudvari–Hízóföld burial ground and its micro region, which could indicate the presence of a power center. In such sites, the high number of these artifacts supports the interpretation of archery equipment as weapon rather than tool.

b) Complexity of single arrowhead interpretations: The evaluation of burials with a single arrowhead is indeed complex. Studies on the burial customs of the 10^th^-century-CE Hungarians (e.g., Tettamanti 1975) have noted the presence of sharp and/or piercing iron object in some graves, placed in a position (e.g., blade facing vertically upward/downward or toward a body part of the deceased) that suggests a purpose other than weaponry, possibly related to superstitious beliefs (e.g., protecting the dead from evil spirits or the living from the dead). Single arrowheads are among these objects, making the interpretation of these burials challenging, especially without broader archaeological context. Furthermore, bioarcheological analysis focusing on the sex determination, lifestyle, and pathological conditions was conducted only on a smaller extent, which is crucial for properly evaluating the function or symbolism of these artifacts. For instance, in the Sárrétudvari–Hízóföld cemetery, burial No. 202 contained two arrowheads placed near the ankle and on the right foot of the deceased, with their tips facing the body. Paleopathological analysis conducted by György Pálfi revealed severe changes in the cranial and postcranial skeleton, particularly the foot bones, caused by leprosy (Pálfi 1991). It is unlikely that the arrowheads were placed in the burial for the same reasons as in graves containing complete archery gear or melee weapons. Instead, the artifact type and its position suggest a connection with the presence of a disease that severely affected the body, making it more likely that these arrowheads served as amulets.

We aim to collect and study all these cases in greater detail in the future. However, without extensive archaeological and bioarchaeological data, we cannot dismiss the earlier interpretations that denied the identification of single arrowheads as weapons in female and sub-adult burials. Consequently, while we appreciate the Reviewer’s suggestion to use a more neutral term, we believe the term ‘first-known with weapon’ remains appropriate within the context of the 10th-century-CE Carpathian Basin archaeology.

References:

Pálfi, Gy. (1991). The First Osteoarchaeological Evidence of Leprosy in Hungary. *International Journal of Osteoarchaeology*, *1*, 99–102.

Révész, L. (2020). *A 10–11. századi temetők regionális jellemzői a Keleti-Kárpátoktól a Dunáig*. Szegedi Tudományegyetem Régészeti Tanszéke–Magyar Tudományos Akadémia Régészeti Intézete–Magyar Nemzeti Múzeum–Martin Opitz Kiadó.

Schulze-Dörrlamm, M. (2023). Spuren der Ungarneinfälle des 10. Jahrhunderts in das Ostfrankenreich. In T. Wozniak & C. Bley (Eds.), *1100 Jahre Quedlinburg. Geschichte - Kultur - Welterbe* (pp. 46–61). Michael Imhof Verlag.

Tettamanti, S. (1975). Temetkezési szokások a X–XI. sz.-ban a Kárpát-medencében. *Studia Comitatensia. Tanulmányok Pest Megye Múzeumaiból*, *3*, 79–124.

**Reviewer: As the authors themselves also indicate in the discussion, archery equipment is likely to have been part of a toolkit that allowed also for defense, and other indications are also present for women in this society to have been skilled horseriders as there have also been numerous indications of women from this time period being buried with horse gear. Therefore, horse-riding and using bow and arrow is definitely a possible component of the female lifestyle for this time period and population. It is however absolutely the case, as the authors describe, that this seems to be a rarer occasion where the skeletal remains can be interpreted with confidence on being female, and have a combination of a bow plate and an arrowhead. This in combination with the healed pathological descriptions on the women having had an active lifestyle, is to me convincing.**

Response: We greatly appreciate the Reviewer’s recognition of the significance of the findings and their agreement with our interpretation of the evidence.

**Reviewer: Small terminology note: The authors refer to the bows of this timeperiod as compound bow. I think this is a translation issue, as it should be composite bow. The term "compound bow" is more commonly used to refer to specific type of modern bow that has a system of pulleys and cables that help in drawing and holding the bowstring. The type of bows discussed in this article should be referred to as "composite bows."**

Response: We are grateful for the Reviewer’s correction. It was indeed a translation issue, and we have replaced the term ‘compound’ with ‘composite’ in the relevant lines (176 and 702 of the original text) of the original text.

**Reviewer: Pathology notes: it is described that ‘the most striking feature’ in the skeleton is the ‘significant lower bone density’. If it is significant that means its measurable, but I don’t see described how this was determined, other than ‘thinning of the bone’ which is quite vague. I am commenting on this because the authors stated themselves that the bone preservation was poor. So how can this be ascertained? It is described that the bone was thin in comparison to other individuals in the series, but no data is reported for the other individuals so it is difficult to say that this is ‘significant’. Specifically in the discussion, it is stated ‘ the presence of osteoporosis…supports the results’. But there are no methods described how osteoporosis is diagnosed. Diagenetic changes in relation to bone loss for investigating age related bone loss, so the clear emphasis on the poor preservation of the bones in relation to sex determination, but no comment on this for bone density, I find a bit concerning.**

**I am giving a few suggestions where methodologies are described how osteopenia or osteoporosis can be measured and described using quantitative methods.**

**Brickley, M. B., & Agarwal, S. C. (2003). Techniques for the investigation of age-related bone loss and osteoporosis in archaeological bone. In Bone loss and osteoporosis: an anthropological perspective (pp. 157-172). Boston, MA: Springer US.**

**Van Spelde, A. M., Schroeder, H., Kjellström, A., & Lidén, K. (2021). Approaches to osteoporosis in paleopathology: How did methodology shape bone loss research?. International Journal of Paleopathology, 33, 245-257.**

Response: We are grateful for the comment and the suggested literature. Unfortunately, during the macromorphological re-evaluation of the skeletal remains of SH-63, the bones could only be examined with the naked eye, and no radiological investigations could be conducted due to time constraints and limited financial support. As a result, standard quantitative methods were not applicable, and the term ‘significant’ was intended to express only the advanced level of changes observed in the bones during the morphological analysis. While *post mortem* damage clearly hinders the definitive diagnosis of the changes, revealing the cross-section and inner structure of certain skull and postcranial elements allowed us to observe the changes described in lines 404–409 of the Results section and illustrated in Figure 4. These features were documented following standard paleopathological references (e.g., Ortner 2003; Waldron 2009).

Thinning of the bones was assessed based on macromorphological examination at sites where the cross-section of the bone was visible due to fragmentation. In addition, measurements on the thickness of the parietals, squamous parts of the temporal, frontal, and occipital bones, and the cortical bone thickness of long bones, where both inner and outer surfaces were intact, generally did not exceed 3 mm (measured with a spreading caliper). For instance, the thickness of the cortical bone of the right humeral diaphysis, shown in Figure 4B, measured approximately at the midshaft, ranged between 1–2 mm – making it challenging to capture a high-quality image. Although these measurements are not standard and do not provide definitive data for evaluation, they support the observations made during macromorphological examination.

Following the Reviewer’s suggestions, we have revised the relevant parts of the Material and Methods, Results, and Discussion and conclusions sections to address the diagnostic limitations and provide a more cautious description. The following changes were made.

In the Material and Methods section, lines 280–281 of the original text now emphasize the use of macromorphological methods for paleopathological investigation:

Original text (lines 280–283 of the original text):

*“We also followed standard osteological and paleopathological research methods and categorization systems (e.g., [89,165,167–169]) to record and identify any pathological changes present on the bones that might have influenced the individual’s lifestyle and the development of activity-related skeletal changes.”*

Revised text (lines 284–288):

“*We also followed standard* *macromorphology-based osteological and paleopathological research methods and categorization systems (e.g., [90, 166, 169–171]) to record and identify any pathological changes present on the bones that might have influenced the individual’s lifestyle and the development of activity-related skeletal changes.*”

In the Results section, lines 404–409 of the original text were rephrased to focus more on the observations made during the macromorphological analysis of SH-63, avoiding assumptions from other cases without a comparative database.

Original text (lines 404–409 in the original manuscript):

“*The most striking feature in the skeleton of SH-63 is the significantly lower bone density compared to similar bones from other individuals in the Sárrétudvari–Hízóföld series. In addition to this, other general conditions were observed in the skeletal remains, such as bone fragility, traces of reduced trabecular system in the vertebrae, an increased diameter of the medullary cavity in the long bones (Fig 4B), and thinning of cortical bone in both the skull and postcranial elements (Fig 4 A & B).*”

Revised text (lines 355–362):

“*A general feature observed in the skeleton of SH-63 is the relatively lightweight nature of the bones. Additionally, other general conditions were noted in the skeletal remains, such as bone fragility, traces of a reduced trabecular system in the vertebrae, an increased diameter of the medullary cavity in the long bones (Fig 4B), and thinning of cortical bone in both the skull and postcranial elements. For instance, the thickness of the parietal bones at the top of the calvaria, measured with a spreading caliper, did not exceed 3 mm (Fig 4A), and the cortical bone thickness of the right humerus, measured approximately at the area of the midshaft, was between 1–2 mm (Fig 4B).*”

In the Discussion and conclusions section, lines 547–557 of the original text were rephrased to address the methodological issues and avoid overinterpretation of the available data. The suggested references were also included:

Original text (lines 547–555 in the original manuscript)

“*Several antemortem features are present on the skeletal remains of SH-63. Firstly, we observed that the bones are much lighter than in the case of other skeletons from the Sárrétudvari–Hízóföld series, possibly due to osteopenia, a loss of bone mass. Particularly, bone fragility, traces of a reduced trabecular system in the vertebrae, an increased diameter of the medullary cavity in the long bones, thinning of cortical bone in both the skull and postcranial elements, and antemortem bone fractures suggest the presence of osteoporosis in the skeleton (e.g., [165,179–182]). The presence of osteoporosis, described as a disease that typically affects older women [165,180], indirectly supports the results concerning the sex determination of SH-63.”*

Revised text (lines 500–516):

“*Several antemortem features are present on the skeletal remains of SH-63. Firstly, we observed the lightweight nature of the bones, which could indicate osteopenia, a loss of bone mass. Specifically, bone fragility, traces of a reduced trabecular system in the vertebrae, an increased diameter of the medullary cavity in the long bones, thinning of cortical bone in both the skull and postcranial elements, and antemortem bone fractures may be associated with the presence of osteoporosis in the skeleton (e.g., [166,174–177]). The presence of osteoporosis, described as a disease that typically affects older women [166,175], could indirectly support the results concerning the sex determination of SH-63. However, the assessment of the registered features is limited for two main reasons. Firstly, diagnosing osteoporosis requires consideration of several methodological factors, including the use of quantitative methods (e.g., radiometry or histomorphometry) [178,179]. As no radiological or histological investigations could be conducted during the analysis of SH-63, there is no such evidence for a definitive diagnosis. Secondly, poor bone preservation complicates the detailed discussion of diagenetic changes in relation to bone loss. Nevertheless, it is unlikely that diagenetic changes alone account for the observed changes, and the presence of pathological conditions, including osteoporosis, should be considered, especially given the antemortem bone fractures found in the skeleton of SH-63.*”

In addition, osteoporosis was deleted from the examples listed in line 656 of the original text.

References:

Ortner, D. J. (2003). *Identification of pathological conditions in human skeletal remains*. Academic Press.

Waldron, T. (2009). *Palaeopathology*. Cambridge University Press.

**Reviewer: Interpretation of the trauma: I find there is some conflicting in the way the authors argue what caused the injuries. On the one hand, it is described that the type of injuries are likely/easily related to osteoporosis, which would be related to aging and bone loss. On the other hand, they interpret it as related to an active lifestyle and that the traumas are consistent with injuries observed on individuals buried with weapons and/or horse riding gear. Are these two interpretations not conflicting?**

Response: We appreciate the Reviewer’s observation regarding the potential conflict in our interpretation of the trauma. While the roles of osteoporosis and an active lifestyle might initially appear contradictory, we believe that the two interpretations complement each other by offering possible explanations for different aspects of the observed changes. On the one hand, the bone loss could have exacerbated the severity of the injuries. According to the literature (e.g., Galloway 2014; Lovell & Grauer 2019), scapular fractures are rare because the muscle layers usually protect the bone. In a young and healthy individual, the same type of incidental injury might have caused less damage or no fracture at all. On the other hand, bone loss alone does not cause injuries; a physical load is necessary for their occurrence. Physical load is commonly associated with accidental falls during walking or other physical activities. Therefore, the nature and location of the injuries (e.g., hip, shoulder, or wrist) are consistent with physical activity. In the case of SH-63, the distribution of traumatic lesions suggests that direct falls on the shoulders during physical activities are the most likely explanation, while the fragility of the bones due to bone loss accounts for the unusual severity of the injuries. This combined perspective helps provide a comprehensive explanation for the observed trauma in SH-63.

References:

Galloway, A. (2014). The upper extremity. In V. L. Wedel & A. Galloway (Eds.), *Broken Bones. Anthropological Analysis of Blunt Force Trauma. Second Edition* (pp. 195–244). Charles C. Thomas.

Lovell, N. C., & Grauer, A. L. (2019). Analysis and interpretation of trauma in skeletal remains. In A. M. Katzenberg & A. L. Grauer (Eds.), *Biological Anthropology of the Human Skeleton. 3rd edition* (pp. 335–384). Wiley- Blackwell.

**Reviewer: I do appreciate the authors writing from line 633 onwards that the interpretation is problematic and there is no over interpretation indeed.**

**Final note – I really appreciate how the conclusion is written up, as the authors clearly avoid over interpretation but at the same time indicate their findings clearly. Its interesting that this research is part of a larger study on DNA from the region – I’ll be looking forward to reading more about the results of the larger studies that can improve the context of this find.**

Response: We are grateful for the Reviewer’s supportive and constructive feedback. We hope that our future research will continue to provide valuable insights and contribute to the broader understanding of this region.

Responses to the comments and questions of Reviewer #3

We greatly appreciate the Reviewer’s thoughtful comments and suggestions, which have significantly helped us improve our manuscript and make it more understandable. Below, we address each question in numerical order.

**Reviewer: 1. In the second paragraph of the Materials and Methods, the authors state that the material from the cemetery under study was initially sexed and then the analyses were repeated using different sexing methods and many of the original sex estimates were confirmed. Were the remaining original males and females deemed of indeterminate sex after re-assessment or were they classified are belonging to the opposite sex from the one originally estimated?**

Response: We are grateful for the Reviewer’s question. Decades after the initial biological sex determination using the method of Éry and colleagues (Éry et al. 1963), an independent investigation was conducted by our colleague, Dr. William Berthon (Berthon 2019), using Brůžek’s morphological method (Brůžek 2002) and DSP2 (Brůžek et al. 2017).

In the majority of unconfirmed cases, the remaining original males and females were deemed of indeterminate sex or non-observable, primarily due to two methodological reasons: a) the results did not meet the required thresholds for a definitive male or female determination; or b) analysis was not feasible due to poor preservation or complete absence of the pelvic bones. The method by Éry and colleagues, which involves the skull, pelvis, and femur, was applicable even when the pelvic bones were unsuitable for analysis.

However, in six cases, opposite results were obtained with one (in four cases; the other method was either non-applicable or yielded an indeterminate result) or with both methods (in two cases) during the reassessment of biological sex. Although we plan to undertake a methodological project to explore the factors behind these discrepancies, this remains a controversial issue for now, as no archeogenetic data is available for these individuals. In addition, these burials contained only general grave goods that were either gender-neutral or absent altogether.

Subsequently, archeogenetic investigations were conducted on a representative number of samples from the series, initially focusing on mitogenome data (Maár et al. 2021) and later on whole genome data (Maróti et al. 2022). In the cases included in these projects, no conflict was detected between the anthropological and archeogenetic sex determination data, except for the sample studied in our manuscript.

References:

Berthon, W. (2019). *Bioarchaeological analysis of the mounted archers from the Hungarian Conquest period (10th century): Horse riding and activity-related skeletal changes*. École Pratique des Hautes Études, PSL University–University of Szeged.

Brůžek, J. (2002). A Method for Visual Determination of Sex, Using the Human Hip Bone. *American Journal of Physical Anthropology*, *117*, 157–168. https://doi.org/10.1002/ajpa.10012

Brůžek, J., Santos, F., Dutailly, B., Murail, P., & Cunha, E. (2017). Validation and reliability of the sex estimation of the human os coxae using freely available DSP2 software for bioarchaeology and forensic anthropology. *American Journal of Physical Anthropology*, *164*, 440–449. https://doi.org/10.1002/ajpa.23282

Éry, K., Kralovánszky, A., & Nemeskéri, J. (1963). Történeti népességek rekonstrukciójának reprezentációja. *Antropológiai Közlemények*, *7*, 41–90.

Maár, K., Varga, G. I. B., Kovács, B., Schütz, O., Maróti, Z., Kalmár, T., Nyerki, E., Nagy, I., Latinovics, D., Tihanyi, B., Marcsik, A., Pálfi, Gy., Bernert, Z., Gallina, Z., Varga, S., Költő, L., Raskó, I., Török, T., & Neparáczki, E. (2021). Maternal Lineages from 10–11th Century Commoner Cemeteries of the Carpathian Basin. *Genes*, *12*, 460. https://doi.org/https://doi.org/10.3390/ genes12030460

Maróti, Z., Neparáczki, E., Schütz, O., Maár, K., Varga, G. I. B., Kovács, B., Kalmár, T., Nyerki, E., Nagy, I., Latinovics, D., Tihanyi, B., Marcsik, A., Pálfi, Gy., Bernert, Z., Gallina, Z., Horváth, C., Varga, S., Költő, L., Raskó, I., … Török, T. (2022). The genetic origin of Huns, Avars, and conquering Hungarians. *Current Biology*, *32*(13), 2858-2870.e7. https://doi.org/10.1016/j.cub.2022.04.093

**Reviewer: 2. The section "Anthropological re-examination of the skeletal remains" is currently too vague; the readers have to check long lists of references to understand what methods the authors used and even then, it is not clear which methods (e.g. for sex estimation) were prioritized over others in cases where different methods produced contradictory results, or how different methods for mechanical stress or pathology were practically implemented in this case give n the partial preservation of the bones (e.g. what proportion of a bone had to be present in order for a condition to be recorded as present/absent?)**

Response: In this section, we intended to convey that multiple biological sex determination methods were considered, though most were not applicable due to the poor preservation of the skeletal remains. Consequently, bone preservation was the primary determinant in selecting the methods, and prioritization was not possible. In response to the Reviewer’s comment, we have rephrased the relevant part of the Methods section to clarify which methods were ultimately applied for anthropological sex determination. Additionally, a sentence from the Results, Sex determination of SH-63 section (lines 367–369 of the original manuscript) has been moved to this part of the Methods.

Original text (lines 274–277 of the original manuscript):

*“As part of our comprehensive investigation, we conducted an anthropological analysis on SH-63, focusing specifically on sex determination and evaluation of activity-related skeletal changes. We applied standard metric and macromorphological methods for sex determination of adult skeletal remains (e.g., [5,9,11–13,15,19,20,23]).”*

Revised text (lines 275–282):

*“**As part of our comprehensive investigation, we conducted an anthropological analysis on SH-63, focusing specifically on sex determination and evaluation of activity-related skeletal changes. Although multiple metric and macromorphological methods for sex determination of adult skeletal remains (e.g., [5,9,11–13,15,19,20,23])* *were considered, only methods examining the morphological traits of the skull were applicable, as the analysis of the coxal bones provided insufficient data for metric or morphological evaluations. Data were recorded based on the standards provided in [5,19], and results were discussed following the descriptions of [5,8,9,19].”*

Similarly, bone preservation was among the main factors limiting the recording and evaluation of supposed activity-related and pathological changes. Generally, we applied a rule commonly found in studies of musculoskeletal system changes or pathological conditions, where at least 50% of the studied entheseal/joint/bone surface has to be present for scoring. However, given the poor bone preservation and the lack of comparative data on females with or without weapons from this population and era, we focused on describing the changes observed on the extant bones rather than scoring them on an ordinal scale. Therefore, we decided to: a) provide full descriptions of the observed changes based on the descriptions of the applied methods instead of providing only scores based on the applied methods, and b) note the presence of changes if the characteristics of the discovered changes fit the definitions of the applied methods (without focusing on severity), even if less then 50% of the given surface was preserved. Furthermore, providing true prevalence data for the entire skeleton proved inappropriate, and we focused on describing the changes observed on the extant skeletal remains for two reasons (in addition to the aforementioned lack of comparative data): a) primarily, we noted ‘presence of changes’ and ‘non-observable’ due to the poor preservation and the scoring rule we followed, and b) as we did not intend to study particular activities (e.g., horse riding or archery), we did not use a standard list of entheses, joints, morphological variants, etc., but instead conducted an overall survey focusing on the pathological and supposed activity-related changes present on the bones.

Following the Reviewer’s comment, the related part of the Methods section was rephrased and extended to clarify how these analyses were implemented.

Original text (lines 277–283 of the original manuscript):

*“In addition, macromorphological methods were used to assess the supposed activity-related skeletal changes of the musculoskeletal system, particularly joint changes [89], entheseal changes [67], morphological variants [4.164], and traumas [165,166]. We also followed standard osteological and paleopathological research methods and categorization systems (e.g., [89,165,167–169]) to record and identify any pathological changes present on the bones that might have influenced the individual’s lifestyle and the development of activity-related skeletal changes.”*

Revised text (lines 282–294):

*“**In addition, macromorphological methods were used to assess the supposed activity-related skeletal changes of the musculoskeletal system, particularly joint changes [90], entheseal changes [68], morphological variants [4,167], and traumas [166,168]. We also followed standard micromorphology-based osteological and paleopathological research methods and categorization systems (e.g., [90,166,169–171]) to record and identify any pathological changes present on the bones that might have influenced the individual’s lifestyle and the development of activity-related skeletal changes.* *Generally, a rule was applied whereby at least 50% of the studied bone surface or part needed to be present for scoring (e.g., [70,82]). However, our primary focus was on describing the changes observed on the extant bones rather than scoring them on an ordinal scale, given the poor preservation of the bones and the lack of comparative data on females with or without weapons from this era. Therefore, changes that met the criteria outlined in the cited studies were noted as ‘presence of changes’ and were described in detail, even if less than 50% of the relevant area was present.”*

**Reviewer: 3. Why did the authors analyze three different elements (petrous bone, tooth, humerus) for aDNA? As expected, they found that the petrous bone performs much better than the others (which in this case did not work at all), so why engage in additional and unnecessary destructive analyses?**

Response: We agree with the Reviewer that, generally, analyzing the petrous bone alone would suffice to obtain the high-quality genetic data and unambiguous results. However, in this case, where correct sex determination is fundamental to all subsequent results and interpretations, we aimed to perform a more extensive analysis. This was also a precautionary step to avoid potential disputes, given the potential for controversy surrounding female burials with weapons and the scrutiny they often face, as many have methodological issues or lack adequate methodological support. A common question is whether both the skull and postcranial skeleton were analyzed. An instructive example is related to a Viking Age case first reported by Hedenstierna-Jonson and colleagues (Hedenstierna-Jonson et al. 2017). After the initial publication, the results were heavily criticized, with even the examination of the proper skeleton being questioned (Price et al. 2019, supplementary documents). They employed a sampling strategy that included both the skull and postcranial skeleton, preventing further disputes.

In our case, we agree with the Reviewer that the tooth from the mandible and postcranial samples did not add significant value to the sex determination results compared to the petrous bone sample. However, the sex determination results were consistent, and this comprehensive approach ensured that all available archaeological, anthropological, and archeogenetic findings and documentation supported the conclusion that this was a single burial of a female individual.

References:

Hedenstierna-Jonson, C., Kjellström, A., Zachrisson, T., Krzewińska, M., Sobrado, V., Price, N., Günther, T., Jakobsson, M., Götherström, A., & Storå, J. (2017). A female Viking warrior confirmed by genomics. *American Journal of Physical Anthropology*, *164*, 853–860. https://doi.org/10.1002/ajpa.23308

Price, N., Hedenstierna-Jonson, C., Zachrisson, T., Kjellström, A., Storå, J., Krzewińska, M., Günther, T., Sobrado, V., Jakobsson, M., & Götherström, A. (2019). Viking warrior women? Reassessing Birka chamber grave Bj.581. *Antiquity*, *93*(367), 181–198. https://doi.org/10.15184/aqy.2018.258

**Reviewer: 4. In the results of the mechanical stress markers, the authors provide very detailed descriptions of the trauma, entheseal changes and arthritis. However, as a result, the reader gets rather lost. It would be helpful to add a skeleton silhouette marking the joints, entheses and other anatomical areas that manifest signs of mechanical stress (e.g. use red for arthritis, blue for entheseal changes, green for trauma). This will help illustrate the distribution of relevant markers.**

Response: We have added the suggested figure (Figure 12) in the Discussion and conclusions section, following the sentence “*In conclusion, numerous changes in the musculoskeletal system have been observed on the bones of SH-63.*” (lines 633 and 634 in the original manuscript). We hope this additional figure will improve readability and visual understanding of the distribution of the skeletal markers.

**Reviewer: Once the above minor issues are addressed/explained, I am happy to see the paper published. It is an original and well contextualized study.**

Response: We are grateful for the Reviewer’s comments and suggestions, which have helped us improve the manuscript and make it clearer for readers.

Responses to the comments of the Academic Editor

We greatly appreciate the Academic Editor’s thoughtful comments and suggestions, which have helped us improve our manuscript and enhance its clarity. Below, we address each of the comments in detail.

**Academic Editor: 1. The authors commendably take a critical approach to the data and emphasise how other explanations may be valid in interpreting the funerary/anthropological/paleopathological context. Converselym, this caution is not matched by the same degree of terminological prudence (as one reviewer notes the presence of an arrowhead and a single bow plate do not constitute sufficient evidence of a female burial with weapons) and the title should be changed, especially by removing the quotation from Tolkien that refers directly to a female warrior character.**

Response: We are grateful for the Academic Editor’s suggestion concerning terminology and potential changes to the title. While we understand the need for precision, based on the written and archaeological sources on the 10^th^-century-CE Hungarians, as well as our findings in this study, we respectfully disagree with the proposed changes. For a more detailed response to the Reviewer’s note, please refer to pages 15–16 of this document.

While the bow and arrow can indeed be considered multifunctional tool used in both hunting and warfare, historical and archaeological scholars consistently agree that the bow and arrow were the primary weapons of 10^th^-century-CE Hungarians. Moreover, their army predominantly consisted of horseback archers, fighting in the traditional style of the nomads of the Eurasian Steppe. Written sources – including accounts from Arabic, Byzantian, and Western European authors – repeatedly describe how the Hungarians primarily relied on their bows in battle, favoring ranged combat over melee combat (e.g., Veszprémy 2010a; 2010b; 2012). These skills were put to the test in numerous military campaigns throughout the 10^th^ century CE. After conquering the Carpathian Basin at the end of the 9^th^ and beginning of the 10^th^ centuries CE, the Hungarians launched well-organized “raids” across Europe. Between 900 and 970 CE, historical sources document 48 mostly successful campaigns (e.g., Veszprémy 2010a, 2012; Révész 2014), which necessitated a standing army and continuous training in both horse riding and archery.

The importance of archery is further reflected in archaeological evidence at the micro-regional level. In most cemeteries, burials containing archery equipment significantly outnumber those with melee weapons. This pattern is especially pronounced in certain regions and sites (e.g., Révész 2020), such as the Sárrétudvari–Hízóföld burial ground and its micro region, which could indicate the presence of a power center (M. Nepper 2002). Given the large number of archery-related finds at these sites, the interpretation of archery equipment as weaponry, rather than merely tools, is well supported. This is especially true for the Sárrétudvari–Hízóföld cemetery, where the highest number of bow fragments from the 10th-century-CE Carpathian Basin were discovered.

In addition, artifacts such as foreign coins (e.g., from Italy and the Byzantine Empire) found in the cemetery suggest that this society was involved in the complex economic system of 10th-century-CE Hungary, where military raids and the taxation of neighboring states played a central role.

We hope that these examples, drawn from written and archaeological sources, will convince the Editor that the interpretation of archery equipment as weapon is justified within the cultural context of 10th-century-CE Hungarian society.

Regarding the quotation in the title, we believe it serves a broader purpose than simply referencing a female warrior character. We have been careful in our manuscript to avoid identifying SH-63 as a warrior, as there is no conclusive data to support this. Instead, we present the fact that an individual initially identified as male — due to the presence of weapon in the grave traditionally associated with males — was, in fact, female. This parallels the situation of the Tolkien character referenced in the title, who was mistaken for a male due to her weapons and attire. Furthermore, the specific phrase, “But no living man am I,” is particularly fitting for the case we present, as SH-63 is indeed a female who lived over a thousand years ago. Thus, we believe that the Tolkien citation effectively symbolizes the broader issue of mistaken identity that our study addresses, where an individual traditionally associated with one gender, due to grave goods, is revealed to be of another. This quotation may serve to engage readers and a broader audience, aligning with our goal, as well as PLOS ONE’s aims of interdisciplinary reach and public engagement. We hope that our reasoning regarding the title and quotation is acceptable.

References:

M. Nepper, I. (2002). *Hajdú-Bihar megye 10–11. századi sírleletei I–II* (Magyarorsz). Déri Múzeum–Magyar Nemzeti Múzeum–Magyar Tudományos Akadémia Régészeti Intézete.

Révész, L. (2014). *The Era of the Hungarian Conquest*. Hungarian National Museum.

Révész, L. (2020). *A 10–11. századi temetők regionális jellemzői a Keleti-Kárpátoktól a Dunáig*. Szegedi Tudományegyetem Régészeti Tanszéke–Magyar Tudományos Akadémia Régészeti Intézete–Magyar Nemzeti Múzeum–Martin Opitz Kiadó.

Veszprémy, L. (2010a). Hungarian raids. In C. J. Rogers (Ed.), *The Oxford Encyclopedia of Medieval Warfare and Military Technology. Volume 2* (pp. 284–286). Oxford Univeristy Press.

Veszprémy, L. (2010b). Hungary - Sources (500-1000). In C. J. Rogers (Ed.), *The Oxford Encyclopedia of Medieval Warfare and Military Technology. Volume 2* (pp. 286–287). Oxford Univeristy Press.

Veszprémy, L. (2012). The military history of Hungary from the first contacts with Europe to Mohács 862-1526. In R. Hermann (Ed.), *Illustrated military history of Hungary* (pp. 13–62). Zrínyi Kiadó.

**Academic Editor: 2. The authors should move the lengthy description of genetic analyses to the supplementary information and reduce the lengthy descriptions of possible methodologies (see those for sex determination). At the same time, more effective and less time-consuming techniques for proteomics of dental enamel are ignored, just as teeth are never analysed.**

Response: We greatly appreciate the Academic Editor’s suggestion regarding the reduction of text and the mention of proteomics techniques. The omission of proteomics in our analysis was intentional, as our team and laboratory specialize in archaeogenetic methods, which are the focus of this investigation. As we did not have the opportunity to conduct proteomic analysis, discussing these techniques without having utilized them would be misleading. In the manuscript, we detailed only the methods that directly impacted our results to ensure clarity for readers unfamiliar with both archaeology, physical anthropology, and archaeogenetics. Although proteomics is mentioned in the Introduction section as one of the most reliable methods in biomolecular sex determination (lines 83–86 of the original text), its absence from our study is thus due to the specialized focus of our laboratory on genetic analyses.

We understand the concern about the potential length and complexity of the methods section. While one reviewer found the detailed description useful, we agree that a balance must be struck between thoroughness and readability. To address this, we considered relocating the more extensive descriptions of genetic analyses to the supplementary information, ensuring that the main text remains focused and accessible. We have also reviewed and condensed descriptions where possible without compromising the scientific validity of our methodology. This way, S1 Text file was created that includes the detailed description of the analysis, and the Methods section was reduced as the followings (lines 295–307 of the revised manuscript):

**“***Archaeogenetic examination of the skeletal remains*

*The pre-PCR procedures were conducted in the specialized ancient DNA facilities of the Department of Genetics, University of Szeged and the Department of Archaeogenetics, Institute of Hungarian Research, Hungary. Mitogenome data of the sample utilized in this study had already been published ([146]), and now we performed shallow shotgun sequencing. Samples were obtained from SH-63 for molecular analysis, including a multirooted tooth from the mandible (referred to as the tooth sample), a sample from the petrous part of the right temporal bone (referred to as the petrosa sample), and a piece from the left humerus (referred to as the humerus sample). A detailed description of the genetic analysis can be found in S1 Text.*

*Biological sex was first assessed with the method described by Skoglund and colleagues [27]. In addition, we used the Rx method first published by Mittnik and colleagues and modified later by de Flamingh and colleagues, which can give accurate results from several thousands of reads mapping to the human genome [30.31].*”

Responses to the comments of the Journal Office

**Journal Office: We note that Figures 1, 2, 3, 4, 5, 6, 7, 8, 9, 10, 11, and 12 in your submission contain [map/satellite] images which may be copyrighted. All PLOS content is published under the Creative Commons Attribution License (CC BY 4.0), which means that the manuscript, images, and Supporting Information files will be freely available online, and any third party is permitted to access, download, copy, distribute, and use these materials in any way, even commercially, with proper attribution. For these reasons, we cannot publish previously copyrighted maps or satellite images created using proprietary data, such as Google software (Google Maps, Street View, and Earth). For more information, see our copyright guidelines:** [**http://journals.plos.org/plosone/s/licenses-and-copyright**](http://journals.plos.org/plosone/s/licenses-and-copyright)**.**

Response: We appreciate your comments regarding copyright issues with the images in our submission.

Figures 2–12, along with the additional figure provided after the revision, consist of original photographs and drawing taken and edited by Luca Kis, one of the authors of this manuscript. Luca Kis is the copyright holder for these images and has granted permission for them to be published in this paper.

In Figure 1B and Figure 2B, the original sources were provided by Zoltán Faur and the Déri Museum of Debrecen. The figures were subsequently created and edited by Luca Kis, as indicated in the respective figure legends and the Acknowledgment section.

In Figure 1A, there is a map, which we have created using QGIS (version 3.16.16), a free and open-source software. We modified the figure legend for Figure 1A to state that it was created in this software as follows:

Original text:

“Fig 1. A) Map of Hungary showing the location of the Sárrétudvari–Hízóföld archaeological site (map edited by Luca Kis).”

Revised text:

“*Fig 1. A) Map of Hungary showing the location of the Sárrétudvari–Hízóföld archaeological site (**created in QGIS (version 3.16.16;* [*https://www.qgis.org/*](https://www.qgis.org/)*), and edited by Luca Kis).*”

In addition, we would like to make the following changes to the Financial Disclosure:

Original statement:

“*This work was supported by the Új Nemzeti Kiválósági Program (grant agreement no.*

*ÚNKP-23-4 -SZTE-650) of the Hungarian Ministry of Culture and Innovation (to BT)*

*and by the Cooperative Doctoral Programme for Doctoral Scholarships 2020 (grant*

*agreement no. 1020404) of the Hungarian Ministry of Innovation and Technology (to*

*LK). Further support was received by European Research Council (ERC) under the*

*European Union’s Horizon 2020 research and innovation programme (grant agreement*

*no. 856453 ERC-2019-SyG) and by the Competence Centre of the Life Sciences*

*Cluster of the Centre of Excellence for Interdisciplinary Research, Development and*

*Innovation of the University of Szeged (to TT). The funders had no role in study design,*

*data collection and analysis, decision to publish, or preparation of the manuscript.*”

Updated statement:

This work was supported by the Új Nemzeti Kiválósági Program (grant agreement no.

ÚNKP-23-4 -SZTE-650) of the Hungarian Ministry of Culture and Innovation (to BT)

and by the Cooperative Doctoral Programme for Doctoral Scholarships 2020 (grant

agreement no. 1020404) of the Hungarian Ministry of Innovation and Technology (to

LK). Further support was received by European Research Council (ERC) under the

European Union’s Horizon 2020 research and innovation programme (grant agreement

no. 856453 ERC-2019-SyG), by the Ministry of Culture and Innovation (grant agreement MCI-670-19/2023/FÁFIN to TT), and by the Competence Centre of the Life Sciences

Cluster of the Centre of Excellence for Interdisciplinary Research, Development and

Innovation of the University of Szeged (to TT). The funders had no role in study design,

data collection and analysis, decision to publish, or preparation of the manuscript.

In the revised version of our manuscript, we have made every effort to incorporate all of the reviewer’s suggestions and have carefully address their concerns. We hope that this updated version is now suitable for publication in PLOS ONE.

Thank you again for your guidance and the reviewers’ insightful and constructive comments. We greatly appreciate your editorial work and consideration.

Yours sincerely,

Dr Balázs Tihanyi, PhD

Corresponding author

Department of Biological Anthropology, University of Szeged

Közép fasor 52, H-6726, Szeged, Hungary

Email: [balazs0421@gmail.com](mailto:balazs0421@gmail.com)

Tel: +36 30 718 0962

Department of Archeology, University of Szeged

Egyetem utca 2, H-6722 Szeged, Hungary

Department of Archaeogenetics, Institute of Hungarian Research

Andrássy út 64, H-1062, Budapest, Hungary
